# Lag penalized weighted correlation for time series clustering

**DOI:** 10.1186/s12859-019-3324-1

**Published:** 2020-07-17

**Authors:** Thevaa Chandereng, Anthony Gitter

**Affiliations:** 1 Department of Biostatistics and Medical Informatics, University of Wisconsin-Madison, Madison, WI USA; 2 Morgridge Institute of Research, Madison, WI USA; 3 Department of Statistics, University of Wisconsin-Madison, Madison, WI USA

**Keywords:** Unsupervised learning, Temporal alignment, Hierarchical clustering

## Abstract

**Background:**

The similarity or distance measure used for clustering can generate intuitive and interpretable clusters when it is tailored to the unique characteristics of the data. In time series datasets generated with high-throughput biological assays, measurements such as gene expression levels or protein phosphorylation intensities are collected sequentially over time, and the similarity score should capture this special temporal structure.

**Results:**

We propose a clustering similarity measure called Lag Penalized Weighted Correlation (LPWC) to group pairs of time series that exhibit closely-related behaviors over time, even if the timing is not perfectly synchronized. LPWC aligns time series profiles to identify common temporal patterns. It down-weights aligned profiles based on the length of the temporal lags that are introduced. We demonstrate the advantages of LPWC versus existing time series and general clustering algorithms. In a simulated dataset based on the biologically-motivated impulse model, LPWC is the only method to recover the true clusters for almost all simulated genes. LPWC also identifies clusters with distinct temporal patterns in our yeast osmotic stress response and axolotl limb regeneration case studies.

**Conclusions:**

LPWC achieves both of its time series clustering goals. It groups time series with correlated changes over time, even if those patterns occur earlier or later in some of the time series. In addition, it refrains from introducing large shifts in time when searching for temporal patterns by applying a lag penalty. The LPWC R package is available at https://github.com/gitter-lab/LPWCand CRAN under a MIT license.

## Background

Time series data are collected extensively to study complex and dynamic biological systems [1, 2]. Tracking the levels of biological molecules such as genes and proteins over time can reveal interactions among them [1] and inform treatment decisions in various diseases [3]. Temporal or longitudinal data are important across multiple disciplines (for example, finance, engineering, and medicine), but biological time series datasets are often shorter than those in other domains. Typically, separate experiments are required for each timepoint, which limits the number of timepoints collected.

Similarity in gene expression patterns can correspond to similarity in biological function, which helps direct future research [4]. Countless clustering algorithms group data points with similar characteristics, but the meaning of “similar” is inherently subjective and application-specific [5]. In time series datasets, similarity must account for the temporal structure. Unlike other data types, observations in time series datasets are dependent on the past. General purpose clustering methods may be able to detect synchronized temporal changes over time but cannot recognize that two entities have the same temporal profile if one is delayed or lagged after the other. In addition, in many cases the timepoints in a biological study are not uniformly distributed over time, and the selection of timepoints is an important aspect of the experimental design [6]. The spacing between timepoints in irregular time series affects the similarity of temporal profiles, especially when allowing lags among the clustered entities.

Many time series clustering algorithms have been introduced to understand the dynamics of biological processes. Some of these clustering approaches are hierarchical, iteratively merging small clusters or dividing large clusters. Others partition entities into clusters, which often requires specifying the number of clusters in advance.

Hierarchical clustering methods, such as clustering with correlation or transformed Euclidean distance for similarity, were a common choice before the proliferation of time series-specific algorithms [4] and continue to be widely used for temporal data [7]. Generic approaches ignore the sequential nature of time series data and give the same clusters even if the timepoints are shuffled, but many temporal hierarchical clustering methods exist as well. Dynamic Time Warping (DTW) aligns timepoints so that the distance between the aligned samples is minimized [8, 9]. Aach et al. introduced a DTW variant that allows aligning the timepoints of one time series to linear interpolations in the other [9]. LEAP allows time delays when constructing co-expression networks [10]. Likewise, Alonso and Peña compute similarity using cross correlation [11]. Short time series (STS) distance computes the rate of change in intensity between adjacent timepoints, but it does not consider lags [12]. Trendy performs segmented regression to summarize temporal expression patterns [13]. TSclust implements multiple clustering approaches including a modified auto-regressive model, numerous distance functions, and a modified wavelet function that accounts for lags [14]. Vilar et al. use forecasting density adopted from auto-regressive models to compute the dissimilarity between time series [15]. TimeClust implements two clustering algorithms, Temporal Abstraction Clustering and Random Walk Models for Bayesian Clustering, developed specifically for short time series [16–18]. Neither of them accounts for lags.

Many partition-based clustering algorithms are available for biological time series data as well. The Short Time-series Expression Miner (STEM) enumerates temporal template profiles and matches genes to them, which works best for short time series (3-8 timepoints) [19]. DynaMiteC [20] clusters genes by fitting them to prototype impulse models [21], but impulses are only one type of common temporal pattern [1]. DynOmics uses the fast Fourier transform to model expression values using mixtures of cyclic patterns [22]. This method also realigns expression values to account for delays but does not treat lagged and unlagged genes differently. Graphical Query Language clusters based on a hidden Markov model [23]. Bar-Joseph et al. turn discrete time series expression data into continuous data using splines [24]. Their clustering algorithm uses the continuous data and expectation maximization to optimize alignment of the temporal data. GEsture is an online graphical tool that takes a hand-drawn curve as input and searches for similar, dissimilar, or delayed gene expression patterns [25]. Other partitioning-based algorithms include a wavelet-based density method using multi-level thresholding [26] and Cluster Analysis of Gene Expression Dynamics, which uses auto-regressive equations [27].

Another category of time series clustering methods is Bayesian models [28–30]. Several of these are built on Dirichlet processes with mixture models that use the temporal information [7, 31]. Dahl proposes a clustering algorithm where genes with similar Dirichlet process mixture components are grouped together and the model is fit using Markov Chain Monte Carlo [31]. McDowell et al. use a Dirichlet process Gaussian process mixture model that determines the number of clusters and models temporal dependencies [7].

**Fig. 1:**
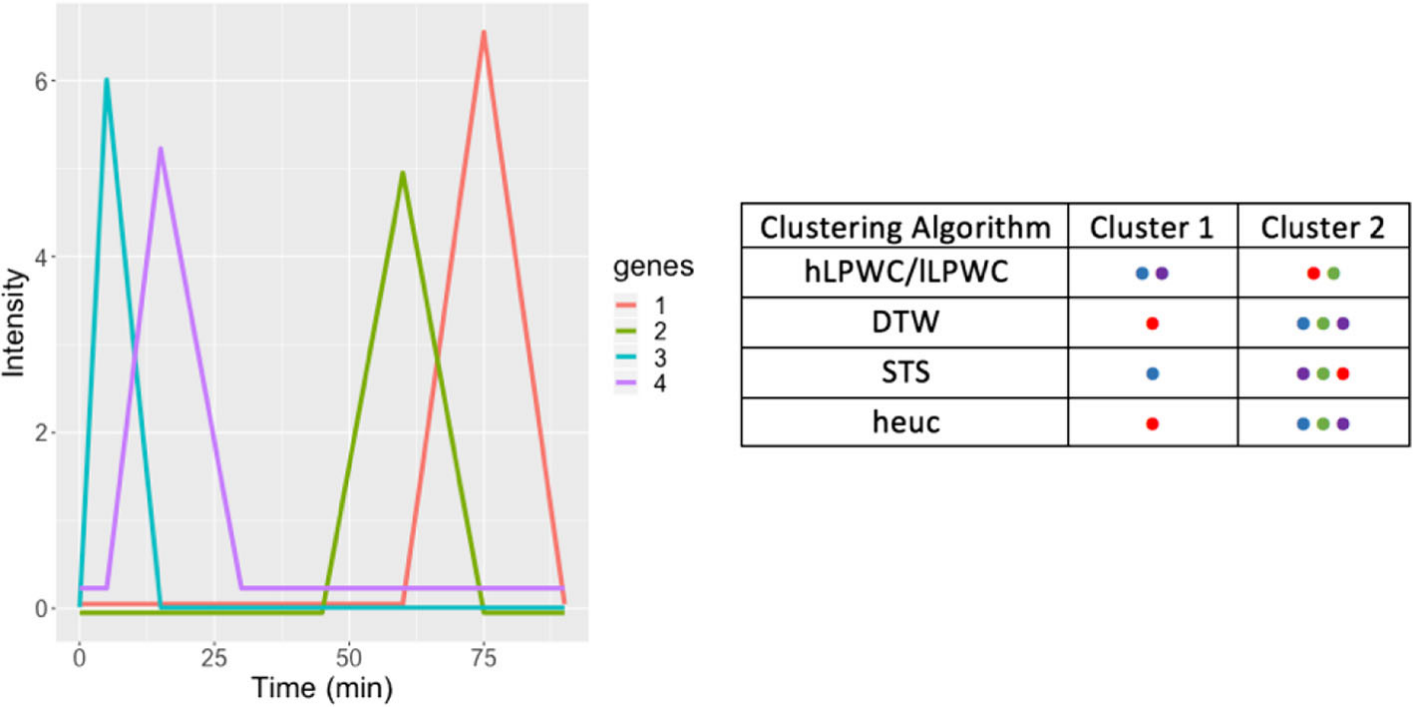
A simple artificial clustering task with four genes and timepoints at 0, 5, 15, 30, 45, 60, 75, and 90 min. Each of the genes has a sharp rise and fall in expression, which occurs at a different timepoint. Genes 1 and 2 both have late spikes and intuitively should be clustered together. Genes 3 and 4 are both early. Several widely used clustering methods group the genes into two clusters, but only LPWC groups the early and late genes correctly. The colored dots in the table represent the different genes

Despite the abundance of clustering algorithms, many popular clustering methods do not have special support for important temporal properties such as lags and irregular timepoints, which we demonstrate with a simple example. Even the methods that do allow lags typically do not treat irregular timepoints differently from regular timepoints. Figure 1 shows how four artificial gene expression profiles are grouped by different clustering methods. This contrived example illustrates desirable properties of a time series clustering algorithm and is not intended to be a formal evaluation. Hierarchical clustering with Euclidean distance (heuc) ignores the timing of the spikes entirely. Two existing time series clustering algorithms, STS and DTW, also fail to group the early and late genes. We introduce a time series clustering algorithm, Lag Penalized Weighted Correlation (LPWC), which captures the delayed responses and the similarity of the early and late genes. LPWC has two modes with a high lag penalty (hLPWC) and low lag penalty (lLPWC).

One of the main contributions of LPWC is a similarity function that accounts for pairs of temporal profiles that occur at slightly different times. This generates a gene-gene similarity matrix that can be used as input for standard similarity- or distance-based clustering methods such as hierarchical clustering. The LPWC similarity score is derived from weighted correlation, but the correlations of lagged temporal profiles are penalized using a Gaussian kernel. The kernel is also used to account for irregular time sampling. We demonstrate the advantages of LPWC over existing general and time series clustering algorithms on a simulated impulse model dataset and case studies on the yeast osmotic stress response and axolotl limb regeneration.

## Results

### Lag Penalized Weighted Correlation overview

The goal of LPWC is to group genes that have similar shapes in their expression levels over time. These shapes or temporal profiles refer to the patterns of increases and decreases in expression. Two genes have similar temporal shapes if the timing of these increases and decreases coincides even if the expression levels are not the same. In order to identify similar temporal shapes that are not perfectly synchronized, LPWC applies a lag operator to re-align the timepoints when comparing two expression profiles. The lag operator compares the timepoints of one expression profile with later timepoints in the other profile. Because the aligned time series can pair measurements that are temporally far apart, LPWC weights the pairs of timepoints to give stronger consideration to those that are close in time.

To assess LPWC, we compared it to other popular clustering algorithms on simulated time series datasets where the true clusters are known and conducted two biological case studies. The yeast osmotic stress response data consist of NaCl-induced osmotic stress phosphorylation samples obtained from mass spectrometry [32]. The axolotl blastema RNA-seq data are collected upon amputating the right forelimb [33]. For the biological case studies, the true clusters are not known, and it is harder to quantitatively evaluate clustering methods. Therefore, we assess whether each clustering algorithm produces clusters with discernible common temporal patterns and makes use of the temporal structure in the data. To assess how the temporal structure is used during clustering, we permute the timepoints.

### Clustering simulated time series data

**Fig. 2:**
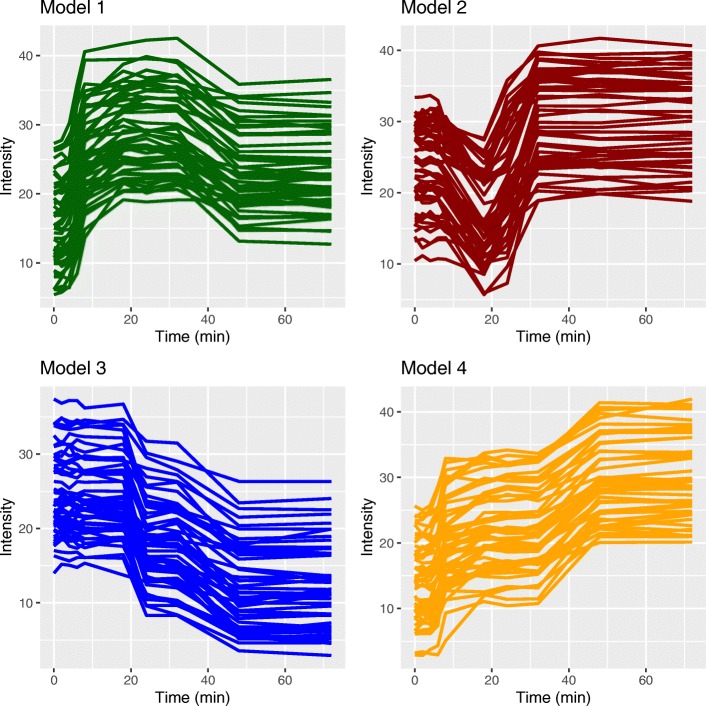
An example of the four patterns simulated using ImpulseDE [21] with low variance. Each model has different characteristics (expression increases and decreases over time) and contains 50 simulated genes

In our primary simulation, each simulated time series dataset contains 200 genes with 10 timepoints at 0, 2, 4, 6, 8, 18, 24, 32, 48, and 72 min. A simulated instance is composed of four distinct temporal patterns with 50 genes per pattern. We repeat the sampling, clustering, and evaluation procedure 100 times in both a low and high variance setting, where the variance controls how similar the simulated genes are to the four reference patterns. Figure 2 shows an example dataset simulated in the low variance setting, and Additional file 1: Figure S1 shows a high variance example. Because the true pattern used to generate each temporal profile is known, the adjusted Rand index (ARI) can be obtained by comparing the true clusters to the cluster assignments produced by different clustering algorithms. ARI of 1 indicates perfect agreement between the true and computed clusters (“Cluster evaluation” section).

We compare LPWC with Euclidean distance with hierarchical clustering (heuc) and kmeans clustering (keuc), Pearson correlation with hierarchical clustering (hcorr) and kmeans clustering (kcorr), DTW with hierarchical clustering, and STS distance with hierarchical clustering (Additional file 1: Section 3). These algorithms include some of the most widely used general clustering approaches as well as two tailored for time series. Instead of using the silhouette method (“Cluster evaluation” section) to pick the number of clusters, all methods return exactly four clusters, the correct number of clusters from the simulation.

**Fig. 3:**
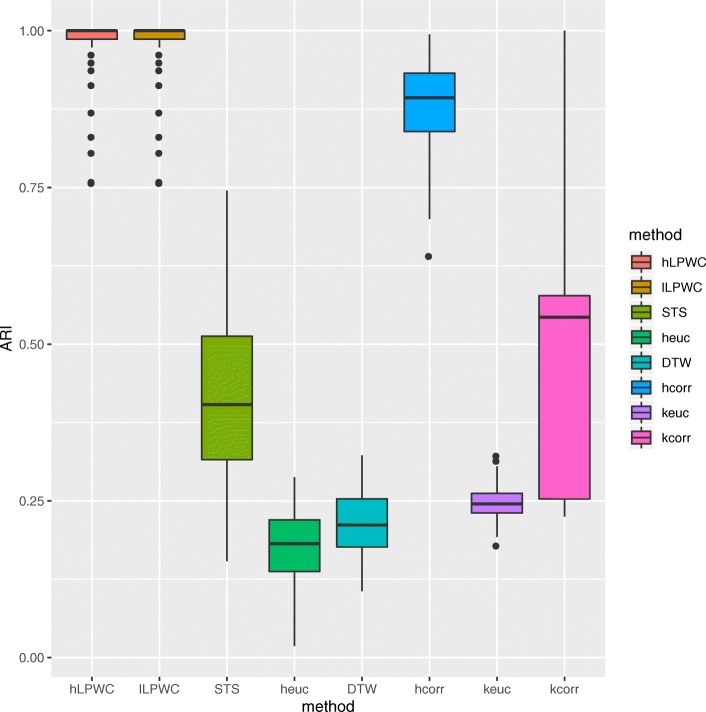
ARI scores with different clustering methods for the low variance simulated impulse data over 100 different simulations

**Fig. 4:**
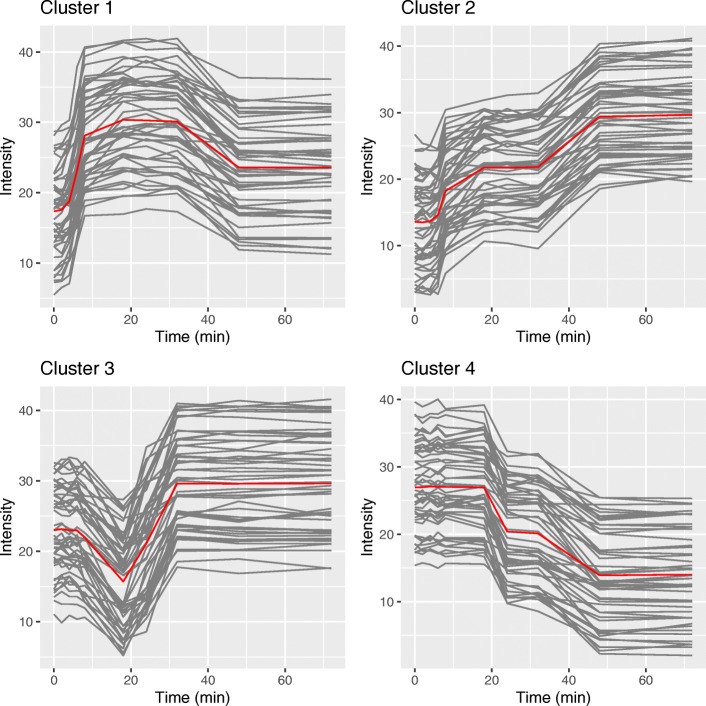
Example lLPWC clusters for the low variance simulated impulse model. The red lines represent the mean intensity values

In the low variance simulation, the two versions of LPWC, hLPWC and lLPWC, outperform all other methods (Fig. 3). The clusters from hLPWC (Additional file 1: Figure S2) and lLPWC (Fig. 4) show that the simulated genes are accurately clustered according to the known assignments. The LPWC ARI scores are close to 1 in almost all of the 100 simulations. The time series clustering methods DTW and STS perform poorly on this task, and hcorr and kcorr are the only other methods that perform reasonably well. This simulation acts as a positive control, demonstrating that LPWC correctly recovers the four temporal expression patterns when we insert moderate offsets in the timing and expression levels.

The high variance simulation is a more challenging clustering task. All methods have median ARI scores less than 0.75 (Additional file 1: Figure S3). LPWC has the best median ARI, but it performs only slightly better than hcorr and kcorr. The correlation-based algorithms (LPWC, hcorr, and kcorr) are more successful than the others because they are robust to the shifts in expression along the y-axis.

LPWC can perform well with both regular and irregular time series data as long as there are sufficient timepoints to characterize the important temporal features. To demonstrate LPWC’s ability to accommodate irregular spacing between timepoints, we extend the simple example from Fig. 1 using the ImpulseDE model. We simulate artificial time series that contain 50 genes with an early spike in expression and 50 with a late spike. In the early group and the late group, half of the simulated genes spike slightly later than the others. We first create a regular time series, sampling the expression from 0 to 72 min every 6 min (Additional file 1: Figure S4). We also construct an irregular time series, sampling at 0, 3, 7, 12, 22, 34, 46, 59, and 75 min (Additional file 1: Figure S5). In both cases, we select the timepoints so that there is a sample before the spike, in the middle of the spike, and after the spike. This enables LPWC to recognize the spike pattern, introduce appropriate lags, and recover perfect clusters regardless of the timepoint spacing (Additional file 1: Figures S6 and S7).

### Yeast osmotic stress response

**Fig. 5:**
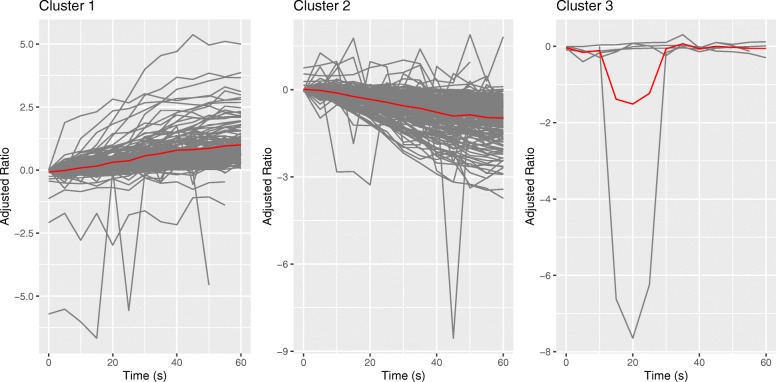
Clusters for the yeast data using the lLPWC algorithm. The y-axis shows the log2 salt/control ratio after subtracting the 0s log2 ratio from all values so all temporal profiles start at 0. The red lines represent the mean adjusted log2 ratios

We used lLPWC to cluster the yeast phosphopeptides in the osmotic stress response dataset into three clusters (Fig. 5 and Additional file 2). Although cluster 3 contains fewer phosphopeptides than the others, this number of clusters was optimal based on our silhouette analysis (Additional file 1: Figure S8). Clusters 1 and 2 were comparable for both lLPWC and hLPWC, with only the smaller cluster 3 showing a notable difference in the mean temporal trend (Additional file 1: Figures S9 and S10 and Additional file 3).

Among the 344 phosphopeptides, there are 33 nonzero lags in lLPWC (Additional file 1: Table S1) and 26 nonzero lags in hLPWC (Additional file 1: Table S2). Although few lags are introduced, they are important in aligning the temporal structure of some phosphopeptides with other phosphopeptides. All clusters exhibit distinct temporal patterns. For visualization purposes, we emphasize these patterns by subtracting the value at 0s from all timepoints before applying the lags and plotting the cluster members and the mean temporal trend (Fig. 5 and Additional file 1: Figure S10). In both lLPWC and hLPWC, most of the phosphopeptides are assigned to clusters 1 and 2 (Additional file 1: Tables S3 and S4). Phosphopeptides in lLPWC cluster 1 demonstrate an overall increasing trend over time. The cluster members are enriched for many broad Gene Ontology (GO) terms related to signal transduction, cellular response to osmotic stress, and actin cytoskeleton organization, which was previously reported to be an important component of this stress response [32] (Additional file 2). Cluster 1 includes the mitogen-activated protein kinase Hog1 and other important proteins in the osmotic stress response pathway such as kinases Pbs2 and Rck2 and transcription factors Msn4 and Sko1. Cluster 2 phosphopeptides show a decrease in phosphorylation over time. The steady increase and decrease trends in clusters 1 and 2 also reflect the major patterns reported by Kanshin et al. [32]. Similar to cluster 1, cluster 2 is also enriched for actin-related terms and general signaling as well as salt and osmotic stress response proteins. These include additional transcription factors Cin5 and Msn2 as well as different phosphorylation sites on Msn4 and Pbs2. Cluster 3, though small, contains a group of phosphopeptides with mostly small changes in phosphorylation over time except for a distinct decrease and increase at 45s. This cluster contains cytokinesis-related proteins.

After permuting the timepoints, the clusters identified should change if the clustering algorithm has detected patterns that depend on the timing. We use ARI to quantify this change (“Cluster evaluation” section). Algorithms that are insensitive to timing will have ARI scores of 1. When comparing the real and permuted data (Additional file 1: Figure S11 and Table S5), nearly all of the hlPWC and lLPWC ARI scores are greater than 0.75. In this dataset, LPWC does not assign many non-zero lags. Therefore, the clustering does not have a strong dependence on the temporal order and is fairly similar with the real and permuted timepoints.

As expected, the four general clustering algorithms (heuc, hcorr, kcorr, and keuc) have ARI scores of 1 because they do not use the temporal information. STS has a low ARI but performs poorly on this dataset. It places all genes into a single cluster, except for two genes that are each assigned to their own singleton cluster (Additional file 1: Figures S12 and S13 and Table S6). DTW does quite well on the yeast data (Additional file 1: Figures S14 and S15). Although three of its clusters are small (Additional file 1: Table S7), the other five contain distinct temporal patterns. The ARI score is also low, showing that DTW does account for the temporal structure in the yeast osmotic stress response data.

### Axolotl blastema

**Fig. 6:**
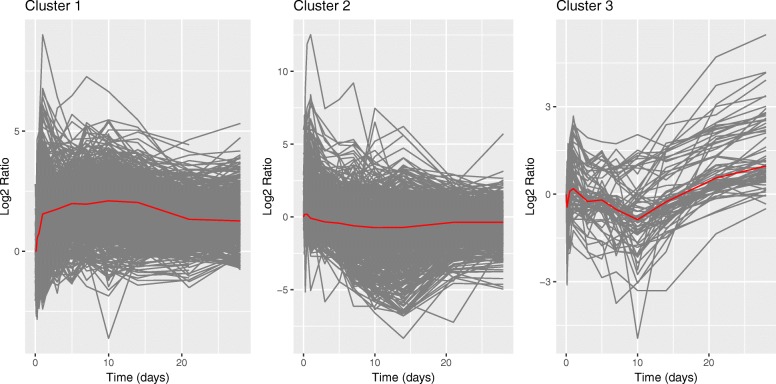
Clusters for the axolotl data using the hLPWC algorithm. The log2 ratio is with respect to the 0 day timepoint. The red lines represent the mean log2 ratios

Figure 6 plots the axolotl blastema gene expression clusters from hLPWC. Three clusters were selected based on the average silhouette width in Additional file 1: Figure S16, and the number of lagged genes and cluster sizes are reported in Additional file 1: Tables S8 and S9, respectively. We added 1 to each expression value before taking the log2 ratios with respect to time 0 days for visualization purposes only. This dampens the extreme fold changes that occur when the initial gene expression level is close to 0, which obscure the temporal trends in each cluster.

The major temporal trends in each cluster are similar for hLPWC (Fig. 6 and Additional file 4) and lLPWC (Additional file 1: Figures S17 and S18, Tables S10 and S11, and Additional file 5). In hLPWC cluster 1, gene expression rapidly then gradually increases from 0 to 14 days, decreases until the 21 day timepoint, and stabilizes afterward. This cluster is enriched for GO terms related to the cell cycle, proliferation, blood vessel development, and wound healing (Additional file 4). The mean cluster 2 trend exhibits down-regulation from 0.25 days until 14 days. The cluster is associated with GO and Kyoto Encyclopedia of Genes and Genomes (KEGG) terms involving ribosomes, RNA-related metabolism, muscle development, and response to oxidative stress. In cluster 3, the mean expression decreases immediately and then rises until day 1, at which point it decreases again until day 10 and then increases for the remainder of the duration. These genes are enriched for type I interferon signaling and other immune processes.

Because hundreds of genes are assigned non-zero lags in the axolotl case study, both hLPWC and lLPWC have low ARI scores when comparing their clusters with the permuted data (Additional file 1: Figure S19 and Table S12). The results are highly dependent on the timing of the expression changes, as desired. As with the yeast case study, the four general clustering methods have ARI scores of 1 or close to 1. STS performs poorly once again. One cluster contains 99% of the genes despite their different temporal characteristics (Additional file 1: Figures S20 and S21 and Table S13). Although DTW is excellent on the yeast dataset, it struggles with the axolotl data (Additional file 1: Figures S22 and S23 and Table S14). The ARI scores are much higher than on yeast, and it places 98% of genes in a single cluster.

## Discussion

LPWC is a generalization of traditional hierarchical clustering with correlation-based similarity. The impulse model simulations illustrate scenarios in which the generalization is most advantageous. When genes are lagged and have different amplitudes than the canonical expression patterns, LPWC still perfectly recovers the correct clusters in almost all of the low variance runs. However, when the simulated genes deviate too much from the canonical patterns, the gene-gene correlations are weaker and LPWC mislabels some genes. When no lags are detected, LPWC is identical to standard hierarchical clustering.

In both the yeast and axolotl case studies, LPWC successfully identifies clusters with unique temporal patterns. LPWC introduces more lags in the axolotl dataset, which may be due to the close timing of the initial timepoints, and the temporal permutation analysis reflects the stronger temporal dependency when there are more lags. The general kmeans and hierarchical clustering algorithms disregard the timepoints by design. STS has performed well in other applications but places nearly all phosphopeptides or genes into a single cluster in both case studies here. DTW, on the other hand, does very well on the yeast application but creates a single dominant cluster on the axolotl dataset. Only LPWC produces useful clusters that depend on the timing for both datasets.

LPWC only considers the expression levels at the observed timepoints and does not interpolate between timepoints or rescale time as in DyNB [34]. Interpolation with line segments [9] or splines [24, 35, 36] makes assumptions about the unobserved behavior between timepoints. Gaussian processes make much weaker assumptions [7], but the kernel function still constrains which types of temporal behaviors and smooth profiles are most likely in between the observed times [37]. In contrast, LPWC assumes that comparing observed values collected at different times is meaningful. This assumption is less likely to hold when the times are far apart, which is why aligned distant timepoints are given a lower weight. If some of the timepoints are very far apart, the time series data could be interpolated with line segments, splines, Gaussian processes, or other approaches before clustering with LPWC.

The LPWC software provides two options to automatically set the parameter *C* that controls the lag penalty. On a new dataset, we recommend running both and inspecting the clustering results to assess whether either of the automatically-selected penalties was effective. If there are no or few lags reported, it may be that the dataset is highly correlated in a synchronous manner or that a lower penalty is needed. The low penalty mode is slower because it runs LPWC repeatedly for multiple values of *C*. Users can also directly manipulate *C* by using the values selected by the high and low penalty modes as a guide. Higher values of *C* impose less of a penalty on far apart timepoints.

Because the main advantage of LPWC is its ability to introduce lags to detect common but unsynchronized temporal patterns, it works best when there are sufficient timepoints to support multiple lags. At least four timepoints are required by LPWC in order to allow one lag. However, if gene *i* and gene *j* are assigned lags of -1 and 1, only two timepoints remain to estimate the correlation. Thus, it is advisable to use LPWC with five or more timepoints. With very short time series we recommend using the high penalty to help avoid spurious correlations. STEM [19], which enumerates temporal patterns, may be preferable for very short time series datasets without delayed responses. DTW [8, 9] has been used extensively in the financial industry for long time series datasets. LPWC can also be applied to long time series, which have sufficient timepoints available to compute reliable correlations even when larger lags are applied. However, the default lag penalties often prevent large lags from being introduced, so *C* may need to be increased beyond the low penalty default. LPWC does not search for common short temporal patterns in a pair of long time series. Rather, it identifies shared trends between the entire prefix of one time series and the entire suffix of another.

Euclidean distance-based or correlation-based similarity measures can be used for clustering. These approaches emphasize different types of temporal shapes. Correlation reveals the trends in the data, whereas Euclidean distance captures the difference in magnitude of expression levels or fold changes. The preference for one over the other is subjective. We prefer correlation for LPWC because it can be applied directly to the original expression levels without computing fold changes with respect to the initial timepoint. Distance-based time series clustering often requires computing these fold changes so that genes are grouped based on their temporal patterns instead of their average expression level, but this effectively drops one of the timepoints because the variation at the initial timepoint is ignored.

## Conclusions

LPWC is designed to capture temporal structure when clustering biological datasets, capable of modeling irregularly-sampled timepoints and detecting delayed responses. It uses lags to align temporal gene expression profiles and weighted correlation to account for irregular sampling. The similarity scores of lagged genes are penalized in order to prefer synchronized temporal patterns and correlations that are computed using a greater fraction of the timepoints. The choice of lags is important because the observations at the beginning and end of the time series are dropped when comparing aligned lagged genes. Therefore, the default parameters are conservative in terms of how many lags are allowed, which is why only a small fraction of phosphopeptides or genes are lagged in our case studies.

Currently, LPWC only accepts a single dataset with one common set of timepoints. In high-throughput biological assays like mass spectrometry and RNA-seq, the timepoints sampled are homogeneous because all proteins or genes are measured with a single experiment at each timepoint. One future direction would be to support clustering multiple related biological datasets with different timepoints. There are also opportunities to better approximate the NP-complete Lag Optimization problem (“Optimal lag optimization” section) and estimate the default value of *C*, which controls how many lags are introduced.

## Methods

### Lag Penalized Weighted Correlation

The LPWC algorithm is composed of three steps: choosing lags for each gene, computing the similarity matrix for all gene pairs, and running standard hierarchical clustering. The best lags are selected by maximizing the sum of the similarities for each gene with respect to all other genes. The maximum possible gene-gene similarity decreases as the lag in the aligned timepoints increases because we prefer to recover temporal behaviors that are synchronized or close in time. In addition, we have less confidence in the similarity of the temporal shapes when they are computed with shorter temporal subsequences. The final correlation-based similarity is computed once the lags for all genes are fixed.

**Fig. 7:**
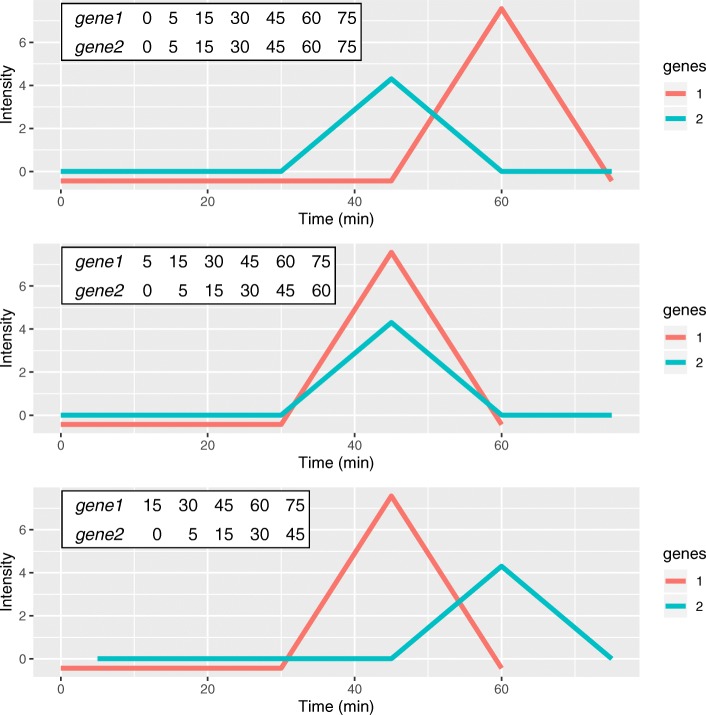
An example of the effects of applying different lags to genes 1 and 2. The three panels show aligned expression vectors *Y*_1_ and *Y*_2_ and aligned timepoint vectors *T*_1_ and *T*_2_. The lagged timepoint vector indices involving *NA* values are dropped from the tables. Top: with no lags, *X*_1_=0 and *X*_2_=0, the temporal profiles of genes 1 and 2 are not aligned so the gene pair will have a low LPWC similarity score. Middle: with lags *X*_1_=−1 and *X*_2_=0, the patterns are aligned, and the LPWC similarity score will be high. Bottom: with *X*_1_=−1 and *X*_2_=1, the temporal shapes are once again not aligned, and the LPWC similarity score will be even lower than in the top row because the penalty for introducing lags is applied

LPWC considers a specific type of local alignment between two time series when assessing the best lag for each gene. The prefix of the time series with the larger lag is aligned with the suffix of the time series with the smaller lag (Fig. 7). The timepoints that belong to the aligned prefix and suffix follow a one-to-one mapping such that the *k*th timepoint of the prefix of one time series is paired with the *k*th timepoint of the suffix of the other time series. The other timepoints are truncated and are not included in the alignment. This alignment strategy differs from the global alignment identified by approaches like DTW. DTW computes a many-to-many mapping between two time series that aligns each timepoint with a timepoint or a linear interpolation in the other time series [9]. Therefore, DTW supports not only time shifts but also time stretches.

LPWC’s correlation-based similarity function for each gene pair *i*,*j* is

$$\begin{array}{*{20}l} {} corr_{LPWC}\left(i, j, X_{i}, X_{j}\right) = \exp\left(\frac{- \bar{w}}{C}\right) * corr_{w}\left(L^{X_{i}}Y_{i}, L^{X_{j}}Y_{j}, \exp\left(\frac{- w}{C}\right)\right) \end{array} $$

where *L* is a lag operator, *X*_*i*_ is the lag for gene *i*, *Y*_*i*_ is the temporal expression levels of gene *i*, *C* is a parameter that controls the lag penalty, *w* is a weight vector, and *corr*_*w*_ is a weighted correlation function. The lag *X*_*i*_ is an integer-valued variable that represents the number of indices a temporal profile is shifted forward or backward in time, where positive values represent forward shifts (Fig. 7). The lag operator *L* can be applied to a vector of temporal gene expression levels (*Y*_*i*_) or a vector of timepoints, which we denote as *T*_*i*_ for gene *i*. *L* reduces the effective length of the lagged vector, introducing *NA* placeholder values. For example, if *T*_*i*_=[0,5,15] and *Y*_*i*_=[0.2,1.4,4.5], then for *X*_*i*_=1 we have *L*^1^*T*_*i*_=[*NA*,0,5] and *L*^1^*Y*_*i*_=[*NA*,0.2,1.4]. For *X*_*i*_=−1 we obtain *L*^−1^*T*_*i*_=[5,15,*NA*] and *L*^−1^*Y*_*i*_=[1.4,4.5,*NA*].

Given this lag operator, we can define the weight vector *w* for weighted correlation.

$$w = \left(L^{X_{i}}T_{i} - L^{X_{j}}T_{j}\right)^{2}$$

The vector subtraction is performed after dropping indices where either vector is *NA*. Similarly, in the weighted correlation, defined here generically for input vectors *x* and *y* and weight vector *z*,

$$corr_{w}\left(x, y, z\right) = \frac{\sum_{i} z_{i}(x_{i}-\bar{x})(y_{i}-\bar{y})}{\sqrt{\sum_{i} z_{i}(x_{i}-\bar{x})^{2}\sum_{i} z_{i}(y_{i}-\bar{y})^{2}}}$$

we drop indices that are *NA* in either *x* or *y* (Fig. 7).

The overall penalty for aligning timepoints is derived from the mean weight $\bar {w}$:

$$\bar{w} = \frac{\sum_{r =1}^{|w|} w_{r}}{|w|}$$

where *r* is an index for the elements of the weight vector and |*w*| is the vector length. When *X*_*i*_=0 and *X*_*j*_=0, *w* is the zeros vector and $\bar {w} = 0$. This makes the weights for *corr*_*w*_ the ones vector. Thus, the special case *corr*_*LPWC*_(*i*,*j*,0,0) is the standard (unweighted) Pearson correlation of *Y*_*i*_ and *Y*_*j*_.

Because choosing the optimal lags *X*_*i*_ for all genes is NP-complete (see “Optimal lag optimization” section for a sketch of the proof), we use a heuristic approach. For each gene *i*, we store the score and respective lag with respect to all other genes *j*. The parameter *m* is the maximum lag allowed. It is important to control the maximum lag because lags reduce the number of data points used to calculate the weighted correlation. We compute

$$score_{j} = \underset{X_{i} \in \{-m,..., m\}}{\text{max}} \;\, corr_{LPWC}(i, j, X_{i}, 0) \quad \forall j \neq i$$

$$lag_{j} = \underset{X_{i} \in \{-m,..., m\}}{\mathrm{arg \,max}} \;\, corr_{LPWC}(i, j, X_{i}, 0) \quad \forall j \neq i$$

Then, a best lag $\hat {X}_{i}$ for gene *i* assigned by

$$\hat{X}_{i} = \underset{k \in \{-m,..., m\}}{\mathrm{arg \,max}} \sum_{j \neq i} I(lag_{j} = k) * score_{j}$$

where *I* is an indicator function. This is repeated to select a best lag for all genes.

Upon obtaining the best lags $\hat {X}_{i}$ for all genes, we compute the similarity

$$\begin{array}{*{20}l} {}corr\left(i, j\right) &= corr_{LPWC}\left(i, j, \hat{X}_{i}, \hat{X}_{j}\right)\\ &= \exp\left(\frac{- \bar{w}}{C}\right) * corr_{w}\left(\!L^{\hat{X}_{i}}Y_{i}, L^{\hat{X}_{j}}Y_{j}, \exp\left(\frac{- w}{C}\right)\!\right) \end{array} $$

The similarity measure *corr*(*i*,*j*) can be used directly by a clustering algorithm that requires gene-gene similarities as input. However, LPWC uses hierarchical clustering, which requires a distance measure instead. We know that −1≤*corr*(*i*,*j*)≤1. Thus, we transform the similarities with *dist*(*i*,*j*)=1−*corr*(*i*,*j*) to obtain distances for hierarchical clustering such that 0≤*dist*(*i*,*j*)≤2. We run hierarchical clustering with complete linkage.

### Controlling the lag penalty

Because lags reduce the number of timepoints used for the correlation calculation and biological time series data are typically short already, there is a risk that two lagged expression vectors will have a high correlation score by chance. Lagged correlation clustering without modification does not perform well [22]. Thus, a Gaussian kernel $\exp \left (\frac {-\bar {w}}{C}\right)$ is used to scale and penalize the weighted correlation based on the lags. The parameter *C* controls the width of the Gaussian kernel function and the severity of the penalty. The appropriate *C* is subjective and application-specific. Therefore, instead of choosing one universal default penalty parameter *C*, LPWC implements two data-dependent ways to set *C*: the high and low penalty modes. The high penalty (hLPWC) penalizes lags more, increasing the possibility of setting *X*_*i*_=0 compared to the low penalty (lLPWC), which will set more *X*_*i*_≠0. In addition to these two default options, the user can also specify *C* directly to introduce more or fewer lags.

The overall penalty that LPWC applies to the weighted correlation *corr*_*w*_ is $\exp \left (\frac {-\bar {w}}{C}\right)$, which scales the correlation by a factor between 0 and 1. For the high penalty, we set the mean penalty over all valid positive lags to 0.5 and solve for *C*

$$penalty(C) = \frac{\sum\limits_{l = 1}^{m} \exp\left(\frac{-\bar{w}^{l}}{C}\right)}{m} = 0.5$$

where *m* is the maximum lag and $\bar {w}^{l}$ is the mean of the elements in the weight vector *w*^*l*^=(*L*^*l*^*T*_*i*_−*L*^0^*T*_*i*_)^2^ obtained from comparing the timepoint vector *T*_*i*_ with a lagged version of *T*_*i*_.

For the low penalty, we compute the values of *C* for which *penalty*(*C*) produces penalties between 0.5 and 0.95 with a step size of 0.05. For each of those *C*, we run LPWC and obtain the gene-gene similarity matrix. We choose the *C* for which the gene-gene similarity matrix is the most stable with respect to the similarity matrix from the previous *C*. Stability is computed by subtracting the two gene-gene similarity matrices, squaring the elements, and summing them. The lowest sum squared difference is preferred. Because it sweeps over multiple values of *C*, lLPWC is slower than hLPWC.

### Optimal lag optimization

“Lag Penalized Weighted Correlation” section describes a heuristic approach for selecting the best lag $\hat {X}_{i}$ for each gene. We now formally define the Lag Optimization problem that the heuristic approximates and sketch a proof that the decision version of Lag Optimization is NP-complete. We define Lag Optimization as:

$${\begin{aligned} \underset{X_{i}^{k}, X_{i,j}^{k,l}}{\text{arg\,max}}&{\sum_{i=2}^{N} \sum_{j=1}^{i-1} \sum_{k \in M} \sum_{l \in M} X_{i,j}^{k,l}*s_{i,j}^{k,l}}\\ \text{subject\ to} &{\sum_{k \in M} X_{i}^{k}}{=1\quad}{\forall \enskip i \in \{1,...,N\}}\\ &{\sum_{k \in M} \sum_{l \in M} X_{i,j}^{k,l}}{=1\quad}{\forall \enskip i \in \{2,...,N\}, j \in \{1,...,i-1\}}\\ &{X_{i,j}^{k,l}}{\leq X_{i}^{k}\quad}{\forall \enskip i \in \{2,...,N\}, j \in \{1,...,i-1\}, k \in M, l \in M}\\ &{X_{i,j}^{k,l}}{\leq X_{j}^{l}\quad}{\forall \enskip i \in \{2,...,N\}, j \in \{1,...,i-1\}, k \in M, l \in M}\\ &{X_{i}^{k}}{\in \left\{ 0,1 \right\}\quad}{\forall \enskip i \in \{1,...,N\}, k \in M}\\ &{X_{i,j}^{k,l}}{\in \left\{ 0,1 \right\}\quad}{\forall \enskip i \in \{2,...,N\}, j \in \{1,...,i-1\}, k \in M, l \in M} \end{aligned}} $$

where *N* is the number of genes and *M* is the set of valid lags. We set *M*={−*m*,...,*m*}, where *m* is the maximum lag allowed. The $X_{i}^{k}$ are binary variables that form a one hot encoding of the integer-valued lag variables *X*_*i*_ from “Lag Penalized Weighted Correlation” section. $X_{i}^{k} = 1$ if *X*_*i*_=*k*, and $X_{i}^{k} = 0$ if *X*_*i*_≠*k*. The $X_{i,j}^{k,l}$ are binary variables that are equal to 1 if and only if the lag of gene *i* is *k* and the lag of gene *j* is *l*. That is, $X_{i,j}^{k,l} = 1$ if $X_{i}^{k} = 1$ and $X_{j}^{l} = 1$, and $X_{i,j}^{k,l} = 0$ otherwise. The $X_{i,j}^{k,l}$ variables are defined only for gene pairs *i* and *j* where *i*>*j*. $s_{i,j}^{k,l}$ is a precomputed similarity score for genes *i* and *j* with lags *k* and *l*, respectively. Here, $s_{i,j}^{k,l} = corr_{LPWC}(i, j, k, l)$, the LPWC similarity score.

To outline the proof that the decision version of Lag Optimization is NP-complete, we show that a solution can be verified in polynomial time and that the NP-complete Weighted Maximum Cut problem [38, 39] can be reduced to Lag Optimization in polynomial time. A solution to Lag Optimization consists of an assignment to all binary $X_{i}^{k}$ and $X_{i,j}^{k,l}$ variables. The decision version of this problem considers whether this solution satisfies the constraints above and whether the objective function is greater than or equal to some value *c*. Given an assignment to the $X_{i}^{k}$ and $X_{i,j}^{k,l}$ variables, we can ensure that only one $X_{i}^{k} = 1$ for each *i* in *O*(|*M*|∗*N*) time and that $X_{i,j}^{k,l} = 1$ if and only if the corresponding $X_{i}^{k} = 1$ and $X_{j}^{l} = 1$ in *O*(|*M*|^2^∗*N*^2^) time. Finally, we can compute the objective function value and assess whether it is ≥*c* in *O*(|*M*|^2^∗*N*^2^) time.

Next, we show that Weighted Maximum Cut reduces to Lag Optimization in polynomial time. In Weighted Maximum Cut [38, 39], we are given a weighted graph *G*=(*V*,*E*) with nonnegative weights *s*_*i*,*j*_ for all *e*_*i*,*j*_∈*E*. The objective is to assign the vertices into sets *V*_1_ and *V*_2_. Edges with one vertex in *V*_1_ and the other in *V*_2_ are cut edges. The decision version of Weighted Maximum Cut assesses whether the sum of the weights *s*_*i*,*j*_ for the cut edges is at least *c*.

To reduce Weighted Maximum Cut to Lag Optimization, first define the set of possible lags *M*={1,2}. Then create variables $X_{i}^{1}$ and $X_{i}^{2}$ for each vertex *v*_*i*_∈*V*. $X_{i}^{1} = 1$ corresponds to placing *v*_*i*_ in vertex set *V*_1_, likewise for $X_{i}^{2}$ and *V*_2_. Create $X_{i,j}^{1,1}$, $X_{i,j}^{1,2}$, $X_{i,j}^{2,1}$, and $X_{i,j}^{2,2}$ variables for all *i*>*j*. Set $s_{i,j}^{1,2}$ and $s_{i,j}^{2,1}$ to the edge weight *s*_*i*,*j*_ for all *i*>*j*. Set $s_{i,j}^{1,1}$ and $s_{i,j}^{2,2}$ to 0 for all *i*>*j*. When $X_{i}^{k} \neq X_{j}^{k}$ in a Lag Optimization solution, the pair contributes a weight of *s*_*i*,*j*_ to the objective function. Otherwise, the pair contributes a weight of 0. Because $X_{i}^{k} \neq X_{j}^{k}$ if and only if the corresponding edge is cut, these pairs are the only pairs that contribute a weight of *s*_*i*,*j*_ to the Weighted Maximum Cut objective function. Thus, the objective function value of the constructed Lag Optimization instance equals that of the original Weighted Maximum Cut instance, and the Lag Optimization and Weighted Maximum Cut decisions are identical. In addition, the transformation from the Weighted Maximum Cut instance to the Lag Optimization instance requires *O*(|*M*|^2^∗*N*^2^) time.

### Simulated time series

To test LPWC, we simulated time series gene expression data using an impulse model called ImpulseDE [21]. Impulses are one common type of temporal pattern in gene expression data [1]. An impulse can be represented as a parameterized curve in which each gene has an initial expression level, increases or decreases in response to a stimulus, and then rises or falls to a new steady state level. The impulse model parameters control each expression level, the timing of the expression increases and decreases, and the curvature of the expression changes (Table 1 and Additional file 1: Table S15).

**Table 1:** ImpulseDE parameters for the four models in the low variance setting

Parameters	Model 1	Model 2	Model 3	Model 4	Parameter variation
*β*_1_	0.8	1.2	1.5	1.2	Uniform(0, 0.5)
*h*_0_	7	13	17	4	Uniform(-3, 3)
*h*_1_	20	6	10	12	Uniform(-3, 3)
*h*_2_	14	20	4	20	Uniform(-3, 3)
*t*_1_	5	8	20	6	Uniform(0, 3)
*t*_2_	40	23	40	44	Uniform(0, 3)

*β*_1_ controls the curvature in the model, *h*_0_,*h*_1_,*h*_2_ control the three different expression state levels, and *t*_1_ and *t*_2_ control the time of expression increase and decrease

We used the ImpulseDE parameters to define four canonical gene expression patterns (models) and simulated 50 genes from each pattern by adding random variation to the model parameters. We ran the simulation in a low variance (Table 1) and high variance (Additional file 1: Table S15) setting to assess how the clustering methods perform as the simulated genes deviate more from the canonical patterns. In the low variance setting, the simulated genes resemble the reference patterns more closely so the clustering problem is easier (Fig. 2). However, in the high variance scenario, the simulated genes are more distorted (Additional file 1: Figure S1), making it harder to recover the correct cluster assignments.

To simulate a gene from a canonical pattern, we randomly sampled an additive offset for each of the six ImpulseDE model parameters using the parameter-specific Uniform distributions in Table 1 and Additional file 1: Table S15. These randomly adjust the expression levels, timing, and curvature. Then, we sampled an additional expression level offset from Uniform(0, 20) and added this to the previously sampled values of *h*_0_, *h*_1_, and *h*_2_. This randomly shifts the entire simulated time course along the y-axis. Given the sampled parameters, we generated expression levels using the impulse model at 10 timepoints: 0, 2, 4, 6, 8, 18, 24, 32, 48, and 72 min. Finally, we added Gaussian-distributed noise to the simulated expression level at each timepoint, sampling from N(0, 0.5) in the low variance setting and N(0, 1) in the high variance setting. We repeated the overall simulation procedure 100 times for both the low and high variance settings to assess the clustering performance over many simulated datasets.

In addition, we used ImpulseDE to study clustering with regular or irregular timepoints and two simple canonical patterns: an early spike and a late spike. The early spike and late spike patterns each had 50 genes, which we divided so that 25 genes spiked slightly later than the other 25 (Additional file 1: Table S16). We selected timepoints to include one timepoint in the middle of the spike and the rest before or after the spike. The regular time series sampled 13 timepoints from 0 to 72 min every 6 min (Additional file 1: Figure S4). The irregular time series sampled 9 timepoints at 0, 3, 7, 12, 22, 34, 46, 59, and 75 min (Additional file 1: Figure S5).

We simulated genes from these two canonical patterns using the ImpulseDE parameters in Additional file 1: Table S16 as described above. We included an additional offset from Uniform(0, 10) to the previously sampled values of *h*_0_, *h*_1_, and *h*_2_ and added Gaussian noise sampled from N(0, 0.5). However, unlike the previous simulations, we added the same offset sampled from Uniform(0, 1) to *t*_1_ and *t*_2_ instead of having two independent offsets. This ensures that the duration of the spike is the same for all simulated genes. We again ran the simulation and clustering process 100 times for both the regular and irregular timepoints.

### Cluster evaluation

Cluster evaluation is difficult because the true clusters are not known for real data. The Rand index compares two clustering results [40]. However, to control for randomness and compare clustering scores from clusters of different sizes, the ARI is a more suitable metric [40]. The ARI is 1 for a perfect clustering that matches the true cluster labels. On the other hand, a score close to 0 indicates a poor clustering. We use the ARI to evaluate clusters of the simulated data where the true cluster labels are known.

One way to evaluate time series clustering algorithms without ground truth labels is by assessing how important the temporal information is to the clustering results. We obtain clusters using the original data and then permute the data by randomly reordering the timepoints (the gene expression observations do not change). The permutations destroy the true temporal dependencies in the data. If a clustering algorithm does not use the temporal information, the ARI score when comparing its clusters on the original and permuted data will be close to 1, which is undesirable. In the yeast and axolotl case studies, we repeat the timepoint permutation 100 times for each clustering algorithm and assess the distribution of ARI scores.

Another challenge is choosing the number of clusters, which can be addressed with the silhouette method [41]. This method assesses whether the clusters are cohesive and distinct from one another. We select the number of clusters that maximizes the average silhouette width.

### Case studies

We applied LPWC in two case studies to demonstrate how it can be used to obtain coherent temporal clusters and derive biological insights into dynamic transcriptional and signaling processes. The first captures the rapid phosphorylation response to osmotic stress in yeast [32]. Kanshin et al. obtained mass spectrometry-based phosphorylation samples in NaCl-induced osmotic stress and control conditions, uniformly sampling 0 to 60 seconds post-stimulation every 5 seconds for a total of 13 timepoints. They transformed these into log2 stress versus control fold changes at each timepoint. We clustered the 344 singly phosphorylated phosphopeptides that were reported to have significant dynamic changes and were not missing values at any timepoints.

The second dataset contains time course RNA-seq data from the axolotl blastema after amputating the right forelimb [33]. Stewart et al. studied the transcriptional changes that take place during the transitions from wound healing to dedifferentiation to limb regeneration. They sampled gene expression at 12 timepoints: 0, 3, 6, and 12 hr and 1, 3, 5, 7, 10, 14, 21, and 28 days post-amputation. Unlike the osmotic stress application, there is drastic irregularity between consecutive sampling times. We converted all times to days. Because the axolotl genome had not been sequenced at the time, Stewart et al. mapped axolotl contigs to human transcripts. They processed the data using edgeR [42], comparing each timepoint to the 0 day measurement to obtain the up- and down-regulated genes. A total of 1656 genes were up- or down-regulated at least at one timepoint compared to 0 day. We ran LPWC on their mapped human gene expression data.

### Gene enrichment analysis

We performed gene enrichment analysis of the LPWC cluster members in DAVID 6.8 [43, 44] (Additional file 1: Section 3.2). For the yeast and axolotl case studies, we report GOTERM_BP_FAT [45] and KEGG_PATHWAY [46] terms that are enriched using DAVID parameters Counts = 2 and Ease = 0.05. The terms were further filtered for false discovery rate ≤5%.

## Availability and requirements

**Project name:** Lag Penalized Weighted Correlation

**Project home page:**https://gitter-lab.github.io/LPWC/

**Operating system(s):** Platform independent

**Programming language:** R (≥ version 3.0.2)

**Other requirements:** None

**License:** MIT

**Any restrictions to use by non-academics:** None

## Supplementary information

**Additional file 1** Supplementary figures, tables, and methods.

**Additional file 2** Cluster assignments and DAVID enrichment for yeast lLPWC as tab-delimited text files. The cluster assignment files contain a header row. The id is the phosphopeptide id from Kanshin et al. [32] and Uniprot is the accession number.

**Additional file 3** Cluster assignments and DAVID enrichment for yeast hLPWC as tab-delimited text files. The cluster assignment files contain a header row. The id is the phosphopeptide id from Kanshin et al. [32] and Uniprot is the accession number. There are no enriched terms for cluster 3.

**Additional file 4** Cluster assignments and DAVID enrichment for axolotl hLPWC as tab-delimited text files. The cluster assignment files contain the mapped human official gene symbols and do not have a header row.

**Additional file 5** Cluster assignments and DAVID enrichment for axolotl lLPWC as tab-delimited text files. The cluster assignment files contain the mapped human official gene symbols and do not have a header row.

## Abbreviations

ARI: Adjusted rand index
DTW: Dynamic time warping
GO: Gene ontology
KEGG: Kyoto encyclopedia of genes and genomes
LPWC: Lag penalized weighted correlation
STEM: Short time-series expression miner
STS: Short time series
