# Supplementary material for: Lag penalized weighted correlation for time series clustering
Source: BMC Bioinformatics. 2020 Jul 17;21:21. doi: 10.1186/s12859-019-3324-1 (PMC6966853; doi:10.1186/s12859-019-3324-1)
Supplement: Supplementary file 1 — Additional file 1 Supplementary figures, tables, and methods. [file 12859_2019_3324_MOESM1_ESM.pdf]

Lag Penalized Weighted Correlation for Time Series Clustering: Supplementary Information

Thevaa Chandereng[1, 2, 3] and Anthony Gitter[1, 2]

[1] *Department of Biostatistics and Medical Informatics, University of Wisconsin-Madison, Madison, WI, USA*
[2] *Morgridge Institute of Research, Madison, WI, USA*
[3] *Department of Statistics, University of Wisconsin-Madison, Madison, WI, USA*

1 Supplementary Figures

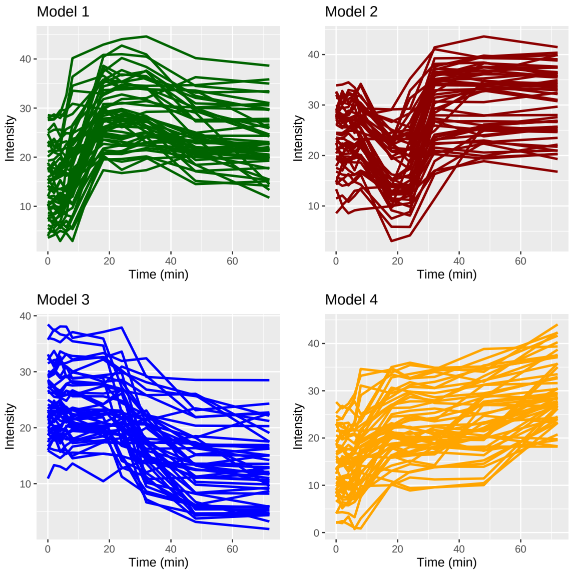

Fig. S1: An example of the four patterns simulated using ImpulseDE with high variance. Each model has different characteristics (expression increases and decreases over time) and contains 50 simulated genes.

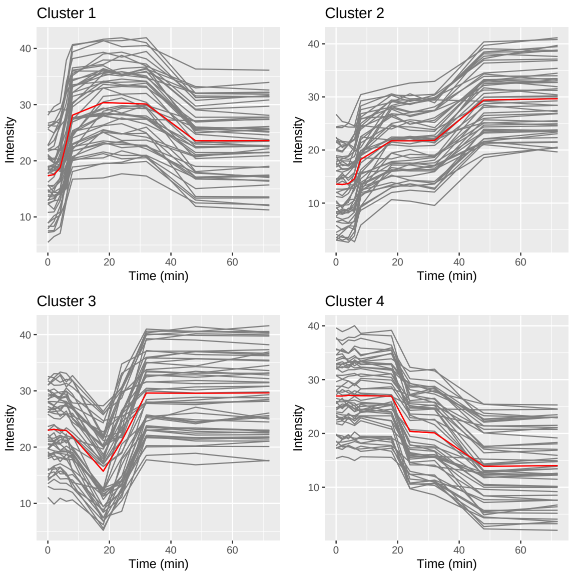

Fig. S2: Example hLPWC clusters for the low variance simulated impulse model. The red lines represent the mean intensity values.

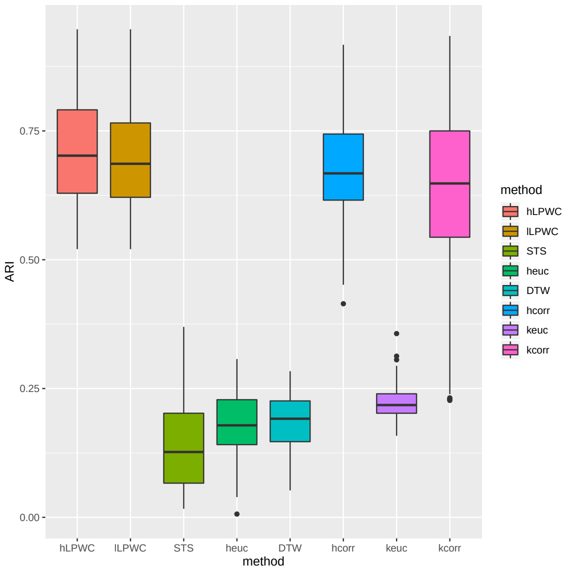

Fig. S3: ARI scores with different clustering methods for the high variance simulated impulse data over 100 different simulations.

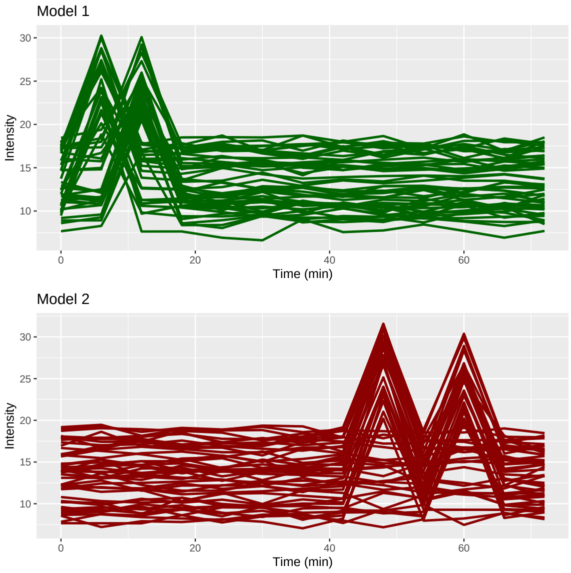

Fig. S4: An example of the two patterns simulated using ImpulseDE with regular time interval sampling. Timepoints are sampled from 0 to 72 min every 6 min. Each model has different characteristics (early or late spikes) and contains 50 simulated genes.

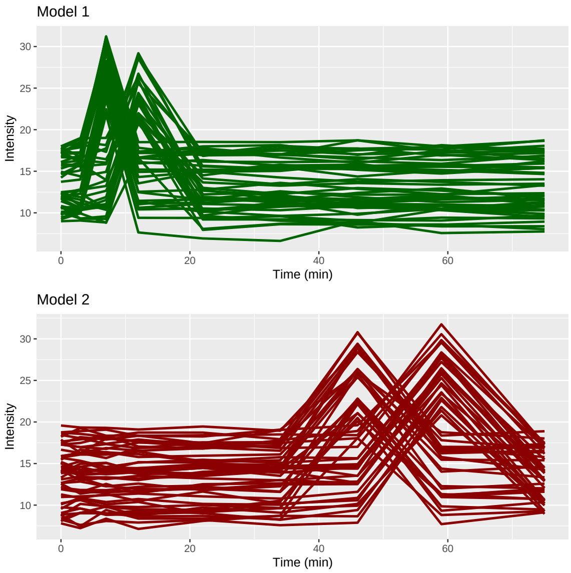

Fig. S5: An example of the two patterns simulated using ImpulseDE with irregular time interval sampling. Timepoints are sampled at 0, 3, 7, 12, 22, 34, 46, 59, and 75 min. Each model has different characteristics (early or late spikes) and contains 50 simulated genes.

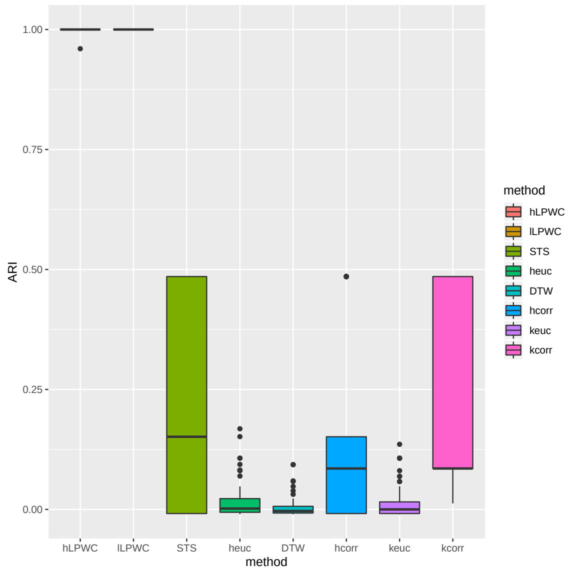

Fig. S6: ARI scores with different clustering methods for the regularly sampled simulated impulse data over 100 different simulations.

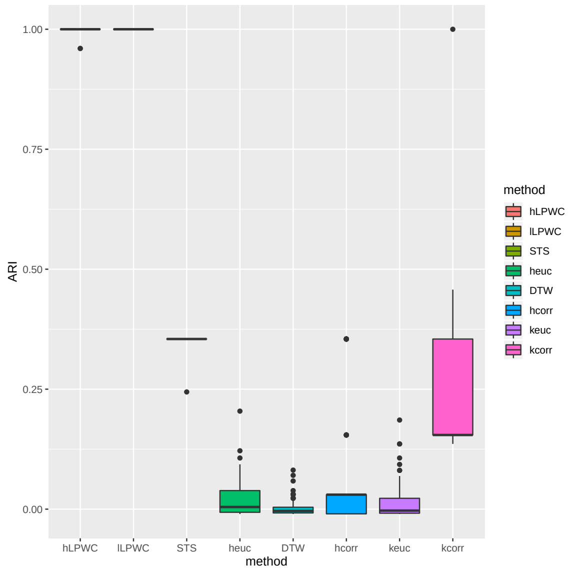

Fig. S7: ARI scores with different clustering methods for the irregularly sampled simulated impulse data over 100 different simulations.

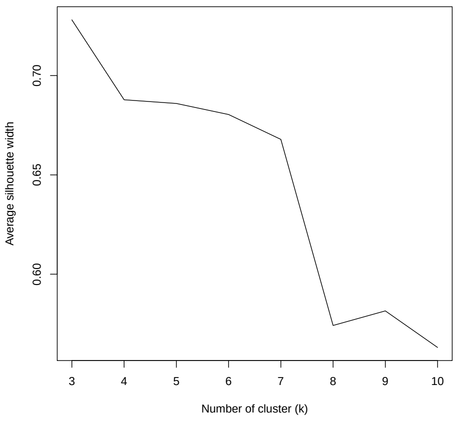

Fig. S8: Average silhouette width for yeast osmotic stress response data using lLPWC with different numbers of clusters. We select three clusters.

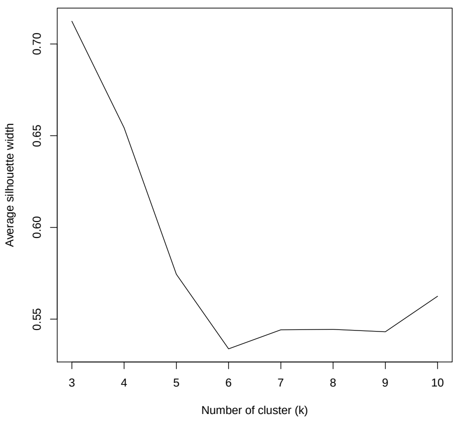

Fig. S9: Average silhouette width for yeast osmotic stress response data using hLPWC with different numbers of clusters. We select three clusters.

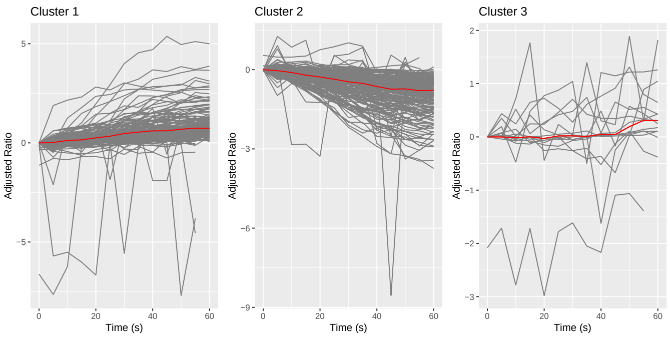

Fig. S10: Clusters for the yeast data using the hLPWC algorithm. The y-axis shows the log2 salt/control ratio after subtracting the 0s log2 ratio from all values so all temporal profiles start at 0. The red lines represent the mean adjusted log2 ratios.

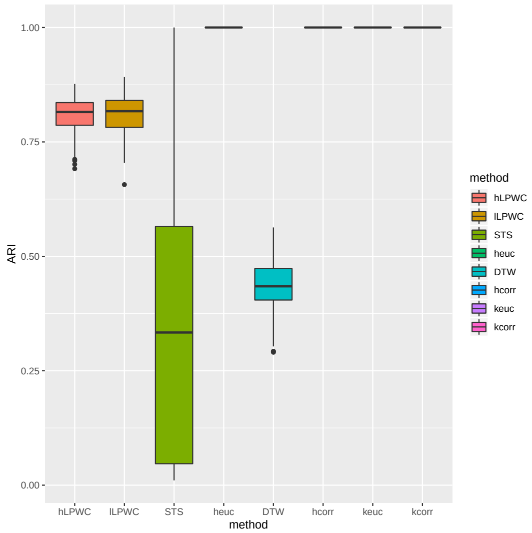

Fig. S11: The distributions of 100 ARI scores using the permuted yeast data.

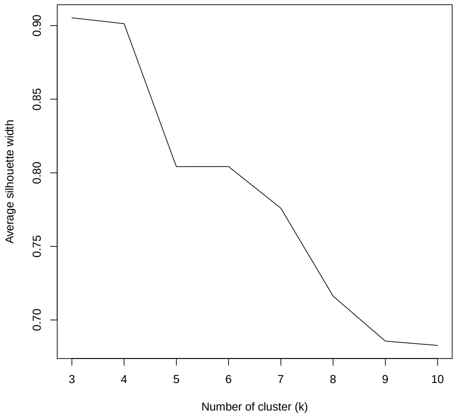

Fig. S12: Average silhouette width for yeast osmotic stress response data using STS with different numbers of clusters. We select three clusters.

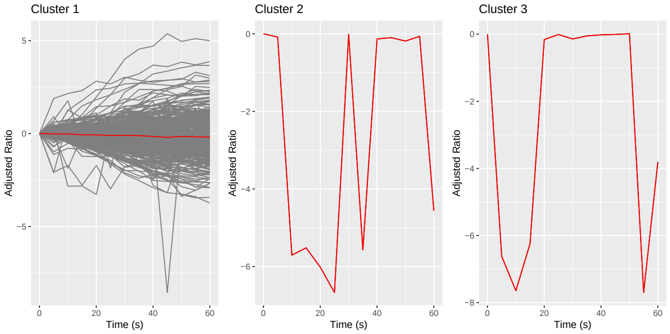

Fig. S13: Clusters for the yeast data using STS. The red lines represent the mean adjusted log2 ratios.

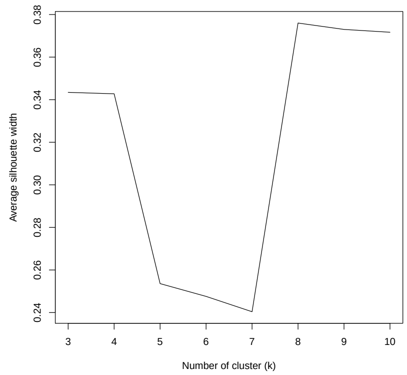

Fig. S14: Average silhouette width for yeast osmotic stress response data using DTW with different numbers of clusters. We select eight clusters.

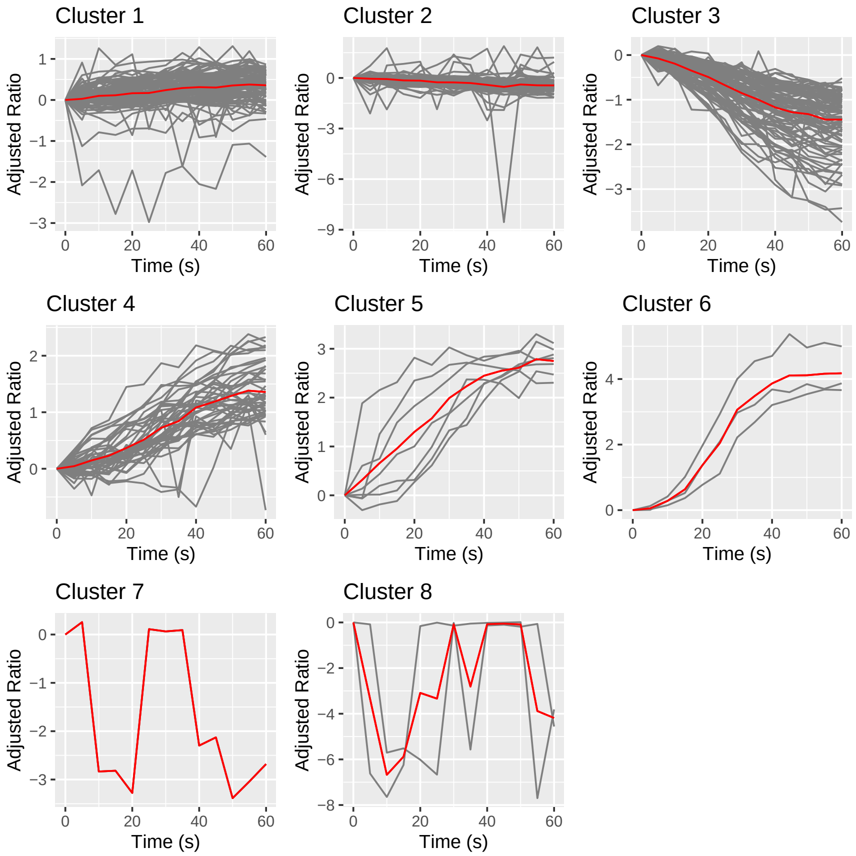

Fig. S15: Clusters for the yeast data using DTW. The red lines represent the mean adjusted log2 ratios.

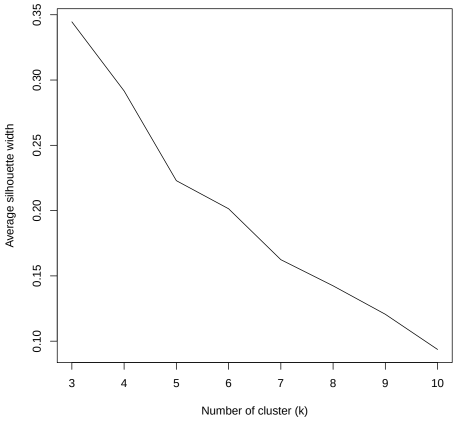

Fig. S16: Average silhouette width for axolotl blastema data using hLPWC with different numbers of clusters. We select three clusters.

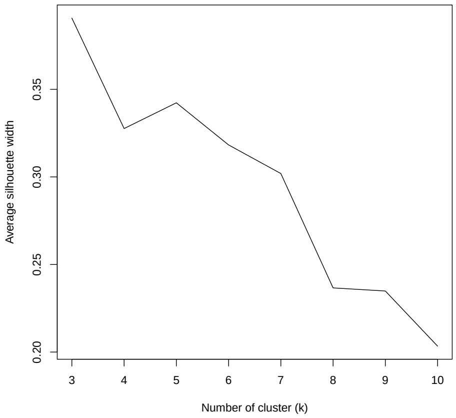

Fig. S17: Average silhouette width for axolotl blastema data using lLPWC with different numbers of clusters. We select three clusters.

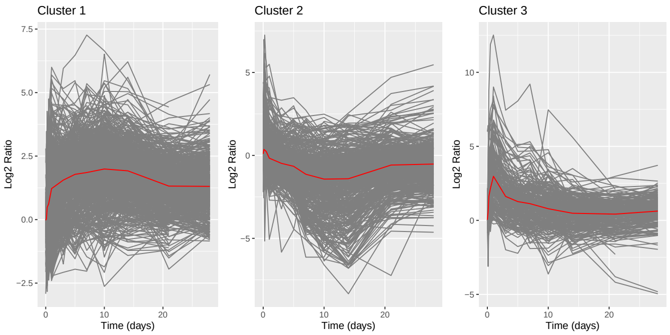

Fig. S18: Clusters for the axolotl data using the lLPWC algorithm. The log2 ratio is with respect to the 0 day timepoint. The red lines represent the mean log2 ratios.

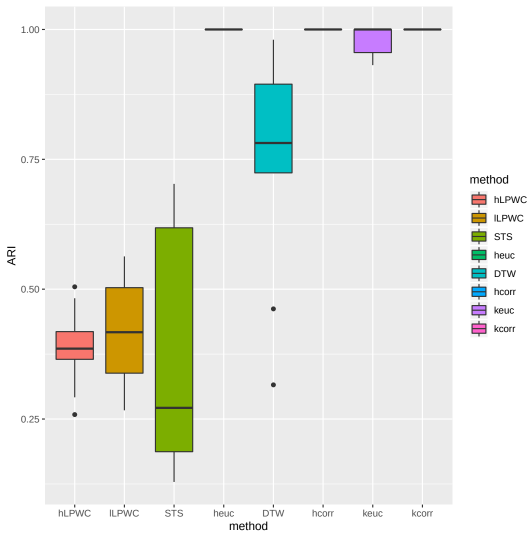

Fig. S19: The distributions of 100 ARI scores using the permuted axolotl data.

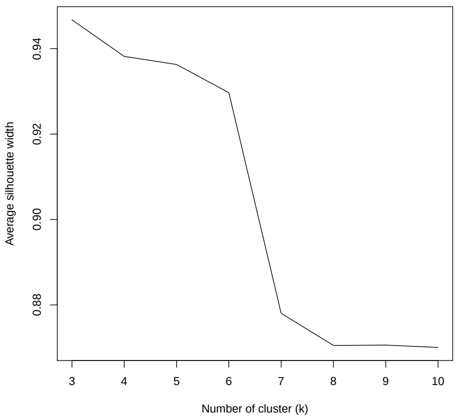

Fig. S20: Average silhouette width for axolotl blastema data using STS with different numbers of clusters. We select three clusters.

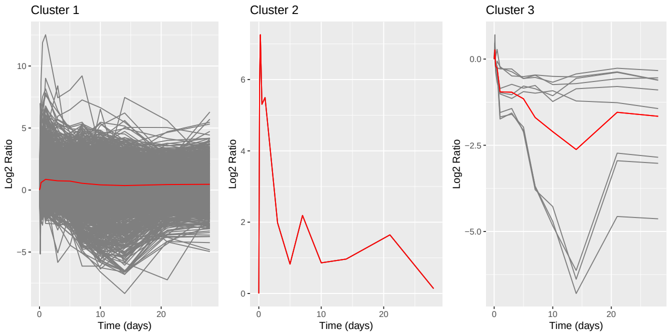

Fig. S21: Clusters for the axolotl data using STS. The red lines represent the mean log2 ratios.

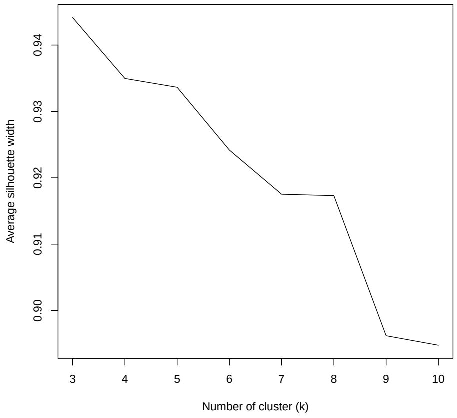

Fig. S22: Average silhouette width for axolotl blastema data using DTW with different numbers of clusters. We select three clusters.

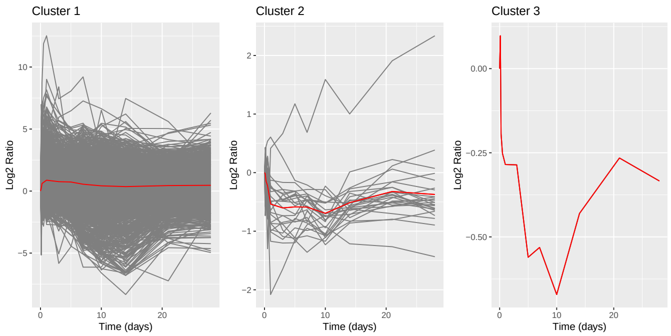

Fig. S23: Clusters for the axolotl data using DTW. The red lines represent the mean log2 ratios.

2 Supplementary Tables

Table S1: Number of phosphopeptides with nonzero lags for lLPWC in the yeast osmotic stress response dataset.

Lags	Number of phosphopeptides
-3	2
-2	6
-1	7
0	311
1	13
2	3
3	2

Table S2: Number of phosphopeptides with nonzero lags for hLPWC in the yeast osmotic stress response dataset.

**Lags**	**Number of phosphopeptides**
-3	1
-2	2
-1	10
0	318
1	11
2	1
3	1

Table S3: Cluster sizes using lLPWC clustering for the yeast osmotic stress response dataset.

**Cluster**	**Number of phosphopeptides**
1	137
2	202
3	5

Table S4: Cluster sizes using hLPWC clustering for the yeast osmotic stress response dataset.

**Cluster**	**Number of phosphopeptides**
1	132
2	198
3	14

Table S5: Mean ARI score for different clustering methods for permuted yeast data. The ARI scores were obtained by comparing the clusters from the permuted data to the clusters from the original data.

k	hLPWC	lLPWC	STS	heuc	DTW	hcorr	keuc	kcorr
3	0.81	0.81	0.38	1.00	0.15	1.00	1.00	1.00
4	0.80	0.81	0.25	1.00	0.17	1.00	1.00	1.00
5	0.79	0.80	0.15	1.00	0.23	1.00	1.00	1.00
6	0.75	0.79	0.08	1.00	0.24	1.00	1.00	1.00
7	0.77	0.80	0.07	1.00	0.25	1.00	1.00	1.00
8	0.78	0.80	0.07	1.00	0.45	1.00	1.00	1.00
9	0.79	0.81	0.07	1.00	0.45	1.00	1.00	1.00
10	0.84	0.82	0.06	1.00	0.45	1.00	1.00	1.00

Table S6: Cluster sizes using STS clustering for the yeast osmotic stress response dataset.

**Cluster**	**Number of phosphopeptides**
1	342
2	1
3	1

Table S7: Cluster sizes using DTW clustering for the yeast osmotic stress response dataset.

Cluster	Number of phosphopeptides
1	95
2	93
3	100
4	43
5	7
6	3
7	1
8	2

Table S8: Number of genes with nonzero lags for hLPWC in the axolotl blastema dataset.

Lags	Number of genes
-2	7
-1	83
0	1428
1	135
2	3

Table S9: Cluster sizes using hLPWC clustering for the axolotl blastema dataset.

Cluster	Number of genes
1	741
2	848
3	67

Table S10: Number of genes with nonzero lags for lLPWC in the axolotl blastema dataset.

Lags	Number of genes
-2	8
-1	121
0	1361
1	156
2	10

Table S11: Cluster sizes using lLPWC clustering for the axolotl blastema dataset.

Cluster	Number of genes
1	736
2	682
3	238

Table S12: Mean ARI score for different clustering methods for permuted axolotl data. The ARI scores were obtained by comparing the clusters from the permuted data to the clusters from the original data. The mean ARI in keuc is not 1 due to the algorithm not converging in 100 random starts and 100 iterations.

k	hLPWC	lLPWC	STS	heuc	DTW	hcorr	keuc	kcorr
3	0.39	0.42	0.38	1.00	0.75	1.00	0.98	1.00
4	0.37	0.39	0.42	1.00	0.84	1.00	1.00	1.00
5	0.40	0.38	0.45	1.00	0.73	1.00	1.00	1.00
6	0.34	0.39	0.38	1.00	0.59	1.00	1.00	1.00
7	0.39	0.39	0.53	1.00	0.87	1.00	1.00	1.00
8	0.36	0.41	0.50	1.00	0.90	1.00	1.00	1.00
9	0.35	0.41	0.50	1.00	0.89	1.00	1.00	1.00
10	0.36	0.35	0.49	1.00	0.89	1.00	1.00	1.00

Table S13: Cluster sizes using STS clustering for the axolotl blastema dataset.

**Cluster**	**Number of genes**
1	1646
2	1
3	9

Table S14: Cluster sizes using DTW clustering for the axolotl blastema dataset.

**Cluster**	**Number of genes**
1	1630
2	25
3	1

Table S15: ImpulseDE parameters for the four models in the high variance setting. β_1 controls the curvature in the model, h_0, h_1, h_2 control the three different expression state levels, and t_1 and t_2 control the time of expression increase and decrease.

**Parameters**	**Model 1**	**Model 2**	**Model 3**	**Model 4**	**Parameter variation**
β_1	0.8	1.2	1.5	1.2	Uniform(0, 0.5)
h_0	7	13	17	4	Uniform(-5, 5)
h_1	20	6	10	12	Uniform(-5, 5)
h_2	14	20	4	20	Uniform(-5, 5)
t_1	5	8	20	6	Uniform(0, 10)
t_2	40	23	40	44	Uniform(0, 10)

Table S16: ImpulseDE parameters for the two models in the regular and irregular timepoint simulations. Each model has two separate components in which the spike occurs slightly earlier (a) or later (b). β_1 controls the curvature in the model, h_0, h_1, h_2 control the three different expression state levels, and t_1 and t_2 control the time of expression increase and decrease.

**Parameters**	**Model 1a**	**Model 1b**	**Model 2a**	**Model 2b**	**Parameter variation**
β_1	5	5	5	5	Uniform(0, 0.5)
h_0	7	7	7	7	Uniform(0, 3)
h_1	20	20	20	20	Uniform(0, 3)
h_2	7	7	7	7	Uniform(0, 3)
t_1	5	9	45	55	Uniform(0, 1)
t_2	8	12	50	60	Same as t_1

3 Supplementary Methods

3.1 Comparison with existing methods

The clustering algorithms used for comparison are Euclidean distance with hierarchical clustering (heuc) and kmeans clustering (keuc), Dynamic Time Warping with hierarchical clustering (DTW), short time series distance with hierarchical clustering (STS), and Pearson correlation with hierarchical clustering (hcorr) and kmeans clustering (kcorr). The DTW distance was computed with the dtw R package version 1.18-1 using the dist function with argument (method = DTW). STS distance was computed with the TSdist R package version 3.4 using the STSDistance function. Pearson correlation with kmeans clustering was computed with the amap R package version 0.8-14 using the Kmeans function. The rest, including hierarchical clustering, were computed using the stats R package version 3.5.0. Euclidean distance was computed using the dist function, Pearson correlation was computed using the cor function, and kmeans was run using kmeans in the R stats package. All methods except STS required the expression data only to perform the analysis. STS also required the timepoints to compute the distance. For hierarchical clustering with Pearson correlation, the correlation matrix was subtracted from 1 to obtain the distances. Both keuc and kcorr were iterated 100 times and randomly started 100 times to achieve convergence. We used LPWC version 0.99.0 for all analyses.

3.2 Gene enrichment analysis

For the yeast cluster DAVID enrichment analysis [1, 2], we used the unique UniProt accessions in each cluster as the query list. Unique identifiers are required because multiple phosphopeptides for the same protein can be co-clustered. The background set was the complete list of yeast UniProt accessions. For the axolotl clusters, we used the mapped human transcripts (official gene symbols) for enrichment analysis in DAVID. The background set was the complete list of human official gene symbols. We used the complete gene or protein lists as backgrounds because our goal was to find common annotations for the clustered genes and proteins to guide cluster interpretation, not to make strong claims about the statistical significance of those annotations. In contrast, Kanshin et al. [3] and Stewart et al. [4] also used DAVID for enrichment analysis but with different background correction strategies. Kanshin et al. removed GO terms that were enriched for the static phosphopeptides from the list of GO terms that were enriched for the dynamically responding phosphopeptides. Stewart et al. considered a background of human genes that had sequence similarity to an axolotl contig and a matching read in the RNA-seq data.

References

[1] Huang DW, Sherman BT, Lempicki RA. Bioinformatics enrichment tools: paths toward the comprehensive functional analysis of large gene lists. *Nucleic Acids Research*, 37(1):1–13, 2008.

[2] Huang DW, Sherman BT, Lempicki RA. Systematic and integrative analysis of large gene lists using DAVID bioinformatics resources. *Nature Protocols*, 4(1):44, 2008.

[3] Kanshin E, Bergeron-Sandoval LP, Isik SS, et al. A Cell-Signaling Network Temporally Resolves Specific versus Promiscuous Phosphorylation. *Cell Reports*, 10(7):1202–1214, 2015.

[4] Stewart R, Rascón CA, Tian S, et al. Comparative RNA-seq Analysis in the Unsequenced Axolotl: The Oncogene Burst Highlights Early Gene Expression in the Blastema. *PLOS Computational Biology*, 9(3):e1002936, 2013.
